# Absence of Repetitive Correlation Patterns Between Pairs of Adjacent Neocortical Neurons *in vivo*

**DOI:** 10.3389/fncir.2019.00048

**Published:** 2019-07-19

**Authors:** Hannes Mogensen, Johanna Norrlid, Jonas M. D. Enander, Anders Wahlbom, Henrik Jörntell

**Affiliations:** Neural Basis of Sensorimotor Control, Department of Experimental Medical Science, Faculty of Medicine, Lund University, Lund, Sweden

**Keywords:** neocortex, pyramidal neurons, spike trains, neurophysiology, circuitry

## Abstract

Neuroanatomy suggests that adjacent neocortical neurons share a similar set of afferent synaptic inputs, as opposed to neurons localized to different areas of the neocortex. In the present study, we made simultaneous single-electrode patch clamp recordings from two or three adjacent neurons in the primary somatosensory cortex (S1) of the ketamine-xylazine anesthetized rat *in vivo* to study the correlation patterns in their spike firing during both spontaneous and sensory-evoked activity. One difference with previous studies of pairwise neuronal spike firing correlations was that here we identified several different quantifiable parameters in the correlation patterns by which different pairs could be compared. The questions asked were if the correlation patterns between adjacent pairs were similar and if there was a relationship between the degree of similarity and the layer location of the pairs. In contrast, our results show that for putative pyramidal neurons within layer III and within layer V, each pair of neurons is to some extent unique in terms of their spiking correlation patterns. Interestingly, our results also indicated that these correlation patterns did not substantially alter between spontaneous and evoked activity. Our findings are compatible with the view that the synaptic input connectivity to each neocortical neuron is at least in some aspects unique. A possible interpretation is that plasticity mechanisms, which could either be initiating or be supported by transcriptomic differences, tend to differentiate rather than harmonize the synaptic weight distributions between adjacent neurons of the same type.

## Introduction

At the macroscopic level, anatomically specific thalamocortical (Jones, 2000) and corticocortical (Malach et al., 1993; Négyessy et al., 2013) connectivity combined with dense intracortical connectivity that gradually tapers off with distance (Fino and Yuste, 2011; Packer and Yuste, 2011) indicate that within a given volume of cortex, the available afferent inputs should be highly similar between adjacent neurons. Pyramidal cells are present in all layers of the neocortex, except layer I (Harris and Shepherd, 2015), but are believed to have particular properties and functions depending on their layer location (Brecht, 2017). Physiological analysis suggests that the intrinsic responsiveness *in vitro* is differentiable between the neuron types (Harris and Shepherd, 2015; Brecht, 2017), and differences in their responsiveness *in vivo* have also been reported (de Kock et al., 2007; Jacob et al., 2012). Further differences between neuron types are believed to exist in their connectivity patterns. Clear differences exist in the output targets of their axons and in the sources of their afferent inputs (Helmstaedter et al., 2007; Harris and Shepherd, 2015). Indeed, based on these and other findings, it has been suggested that there is a canonical microcircuitry in the neocortex, where the subtype and layer identity of the constituent neurons are important determinants of the structure of that microcircuitry (Helmstaedter et al., 2007; Harris and Mrsic-Flogel, 2013; Reimann et al., 2015). Some evidence indicates that between neuron types, the learning processes can show different forms of experience-dependent input plasticity (Jacob et al., 2012), which in turn suggests that the learning rule underlying synaptic plasticity differs between neuron types but is relatively uniform within the neuron type (Holtmaat et al., 2006). A prediction that could be made from this collection of results is that neurons of the same type that are located next to each other should have acquired similar synaptic weight distributions in their afferent inputs and respond to those inputs in a similar way (Ocker et al., 2017), which to some extent seems to be confirmed by connectivity studies (Jiang et al., 2015). In contrast, for the decoding of tactile afferent input patterns in primary somatosensory cortex (S1) neurons, it was recently shown that adjacent neurons differ widely in terms of their decoding performance and that layer location has no predictive value for the decoding performance of the neuron (Oddo et al., 2017).

The correlation patterns in the activity between pairs of cells in the neocortex can provide insights into the physiological network structure. If there are repetitive connectivity motifs in the neocortex, pairs of neurons of the same type would be expected to be connected to the global network in a similar way and thus the correlation patterns between such equivalent pairs should accordingly have common features. Alternatively, if the connectivity with the surrounding network is more unique for individual neurons, the correlation patterns between cell pairs would also be expected to be more unique. So far, this issue has not received much attention. Lampl et al. (1999) made intracellular recordings from pairs of neurons in the visual cortex *in vivo* using two separate patch clamp electrodes. The illustrated raw data suggests that the membrane potential correlations between pairs can have very different shapes. However, these neurons were separated by large distances and therefore expected to receive dissimilar synaptic inputs. Here, we instead wanted to explore the correlation patterns between adjacent neurons that would be expected to receive highly similar synaptic inputs.

Even though the afferent synaptic inputs can be measured directly by dual intracellular recordings, the spike output of a neuron, in general, corresponds to a probability-density function of the intracellular membrane-potential changes, which are induced by the afferent synaptic inputs (Spanne et al., 2014). Hence, the spike output can be considered an approximation of the intracellular potential. In the present study, we take advantage of a technique to record the extracellular spikes from two or more adjacent neurons simultaneously by the same patch clamp electrode. The advantage of this approach is that the simultaneously recorded neurons, because of their overlapping location, can be expected to have a maximal overlap in their anatomically defined afferent synaptic inputs and hence provide a reasonably precise test of the canonical microcircuitry idea. Here, we compare the correlation patterns between pairs of adjacent putative pyramidal neurons recorded primarily in layers III and V and also examine the robustness of these correlation patterns across spontaneous and sensory-evoked activity.

## Materials and Methods

### Surgical Procedures

Adult male Sprague-Dawley rats (*N* = 16, weight 250–380 g) were prepared and maintained under anesthesia with a ketamine and xylazine mixture (20:1). Following isofluorane sedation (2% for 30–60 s), anesthesia was induced *via* an i.p. injection (40 mg/kg of ketamine, 2 mg/kg of xylazine) and maintenance was administered through an intravenous catheter inserted into the right femoral vein (approximately 5 mg/kg ketamine per hour with a continuous infusion). For recording sessions, the level of anesthesia was monitored with a surface electrocorticogram (ECoG) electrode placed in the vicinity of the recording area. The ECoG was characterized by irregular occurrences of sleep spindles, a sign of deep sleep (Niedermeyer and da Silva, 2005). The level of anesthesia was additionally characterized by an absence of withdrawal reflexes to noxious pinches to the hind paw. The decision to run the neuronal recording experiments under anesthesia was motivated by the need to make sure that the mechanical stability of the brain was consistently high throughout the experiments in order to be able to run the long-term *in vivo* recordings necessary. This type of anesthesia has no disruptive effect on the order of neuronal recruitment of neocortical neurons in spontaneous brain activity fluctuations (Up states, recordings obtained using multielectrode arrays in the rat) as compared to the awake condition, which suggests that the physiological structure of the neocortical network may work close to normal (Luczak and Barthó, 2012), even though for example, the global brain state regulation does not. All animal experiment procedures in the present study were in accordance with institutional guidelines and were approved in advance by the Local Animal Ethics Committee of Lund, Sweden (permit ID M118-13).

### Recordings

All recordings were made in the forepaw region of the S1, as estimated by the focus of the local field potentials (between layers III and V) evoked by electrical stimulation of the second digit on the contralateral forepaw. The coordinates of this region were 0.0–1.0 mm rostral 3.5 and 4.5 mm lateral to bregma. Neurons were recorded with patch clamp pipettes extracellularly in the loose patch recording mode. Patch clamp pipettes were pulled from borosilicate glass capillaries to 6–15 MOhm using a Sutter Instruments (Novato, CA, USA) P-97 horizontal puller. The composition of the electrolyte solution in the patch pipettes was (in mM) potassium-gluconate (135), HEPES (10), KCl (6.0), Mg-ATP (2), EGTA (10). The solution was titrated to 7.35–7.40 using 1 M KOH. In order to find neurons, recorded signals were continuously monitored. During slow advancement of the recording electrode (approximately 0.3 μm per second), all the skin stimulation sites were activated with one pulse per second, and any neuronal spike activity encountered was typically recorded from. Dual and triple recordings were obtained from a single patch pipette. All data were digitized at 100 kHz using CED 1401 mk2 hardware and Spike2 software (Cambridge Electronics Devices, CED, Cambridge, UK) and the recording depth from the surface of the brain was annotated. For identification of neuron identity, in addition to depth, we used the nature of the firing, where the occasional presence of doublet or triplet spike firing, but absence of longer bursts or sustained periods of high firing frequency were taken as an indication that all the neurons recorded were pyramidal cells rather than interneurons (Luczak et al., 2009).

### Artificial Tactile Stimulation

The recordings were made in a set of experiments similar to those in Oddo et al. (2017), where the volar side of the second digit of the contralateral forepaw was equipped with four pairs of stimulation electrodes. Through the stimulation electrodes, the rat was then episodically presented with repeatable spatiotemporal patterns mimicking the touching of an object with four different curvatures (in total eight patterns), as described in Oddo et al. (2017). The eight spatiotemporal stimulation patterns were delivered in a pre-defined random order, where the stimulation patterns lasted for less than 340 ms and the consecutive deliveries of the stimulation patterns were separated by 1.8 s. In this relaxation phase, the firing activity of the neuron was then free from external inputs. The measured cell activity was collected both during active stimulation and during spontaneous activity. In the analysis where the two periods were considered separately, a stimulation episode was defined as the onset of the stimulation pattern until 200 ms after its termination to allow a longer period of relaxation from the evoked activity while still maximizing the total time counted as spontaneous activity. Hence the 1.8 s cycle was in this case divided into approximately 540 ms of stimulated activity and 1,260 ms non-stimulated or spontaneous activity.

### Post Processing

**Figure 1:**
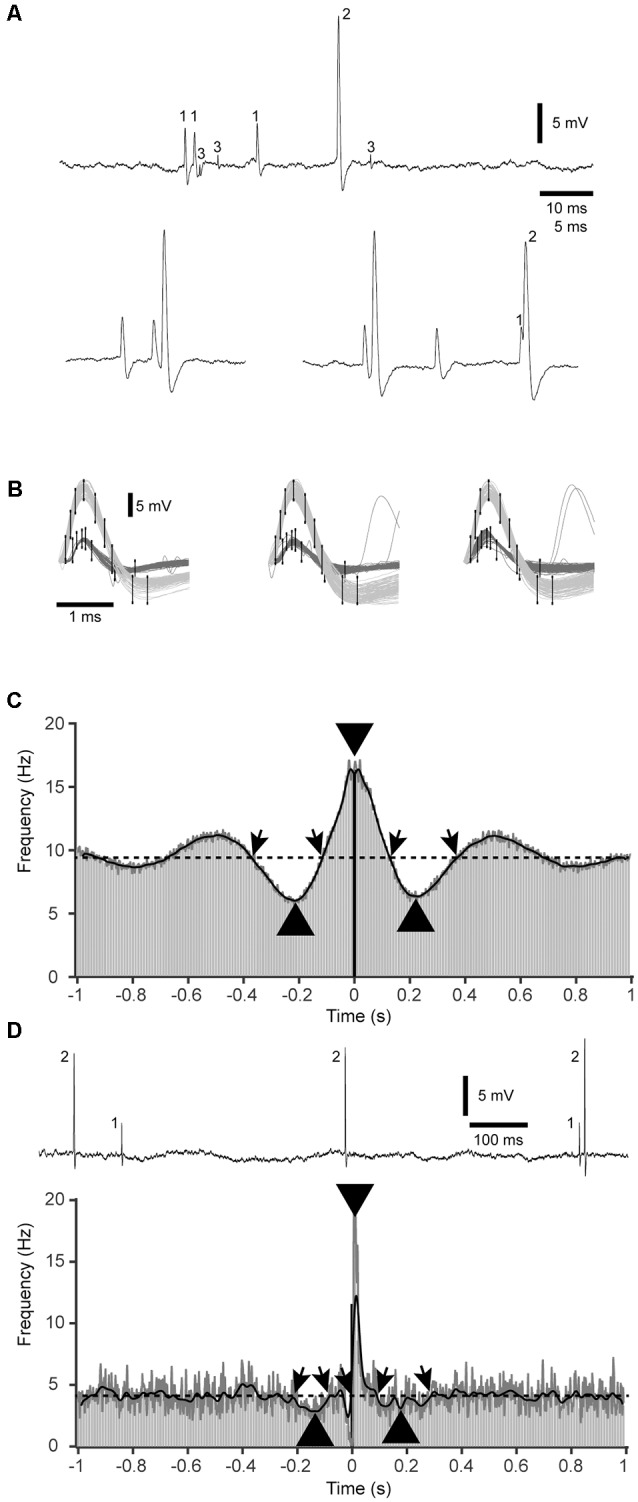
Sample raw data, spike-triggered time histograms and kernel density estimations (KDEs). **(A)** Raw data of a triple neuron recording. Based on the number of recurring spike shapes in the recorded signal, each unitary spike is labeled 1–3. The lower two traces at a higher resolution illustrate a sharp correlation between spikes #1 and #2, and that also near-coincident spikes could be separated. **(B)** Illustration of the principles of operation of the template-based spike identification. Each spike was defined by a set of threshold levels distributed in time, which the signal had to fit in order to count as a spike of aspecific type. In this case, two separate sets of templates were used to identify and separate spikes #1 and #2. **(C)** An example of a symmetric type of correlation between two cells in a pair. Gray bars are the raw data peri-spike time histogram (PSpTH, bin width 10 ms). The bar at time 0 is in black. The black thick line is the SpT-KDE with a standard deviation of 10 ms of the underlying spike times. The thinner dark gray line is a SpT-KDE with a more narrow kernel (standard deviation of 1 ms), not to be confused with the gray bars of the PSpTH. The dashed horizontal line indicates the average baseline activity. Oblique arrows indicate where the SpT-KDE with the wider kernels crossed the average baseline, and was defined as the onsets and offsets of pre-, peri-, and post-trigger deflections in the correlation between the two cells. Thicker arrowheads indicate the peak points of these deflections, with respect to their time and amplitude. **(D)** Raw recording data and correlation pattern plot for another cell pair, in this case with an asymmetric distribution of the peri-trigger deflection and very weak pre- and post-trigger deflections. Note that the raw histogram in this display is overshadowed by the rapidly shifting peaks of the SpT-KDE with the narrow kernel. Note also that the gray bar after time 0 reaches all the way up to the peak identified by the gray SpT-KDE line, whereas the thicker black SpT-KDE failed to fully capture the peak amplitude of this rapid deflection. This was the reason why the narrow-kerneled SpT-KDE was used. This is the same cell pair as in (**A,B**; spikes #1 and #2).

The signal was imported from Spike2 to Matlab, where it was low-pass filtered using a moving average over 50 μs, i.e., a width of five samples. Cellular spikes were identified from the signal (Figure 1A) using tailored template matching routines with manually constructed templates (Figure 1B). As previously described, this method can be used to identify two separate units recorded from the same patch pipette (Bengtsson and Jörntell, 2009; Bengtsson et al., 2013). As shown in Figure 1B, the specificity of the template-based spike identification was high. Each spike template was adapted to identify the same spike in all parts of the recording, as verified by visual inspection of a high number of random raw recording traces (visualized in time-voltage diagrams with a duration of 50–300 ms) in the beginning, the middle and the end of the recording. One means of verification of the accuracy of the spike sorting was an absence of double identification of the same spike by the different templates throughout recordings. The detection of simultaneous and near simultaneous occurrences of two different spikes could be achieved as the template shapes of one spike were subtracted from the signal before the other spike was identified or, alternatively, by manual identification. As in all identification of neuronal spikes, a verification that 100% of the spikes recorded from an individual neuron was also captured in the analysis was impossible to achieve, as there is no ground truth that goes beyond visual identification and even visual identification is not a guarantee of capturing all spikes a given neuron fires. However, our template-based method does not come with any systematic error, and any possible failure in spike identification would therefore not be systematic in the sense that it would affect the shape of the correlation patterns analyzed below. Stimulation artifacts were removed using subtraction of a tailored adaptive template of the artifact signals, to facilitate identification of cellular spikes coinciding with stimulation artifacts.

**Figure 2:**
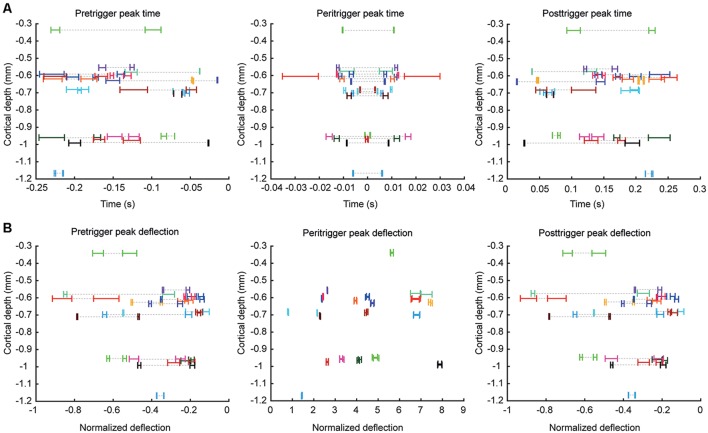
Quantified parameters for comparing the correlation patterns between different cell pairs. For each parameter illustrated, the data of all cell pairs are shown. **(A)** Peak times for the pre-, peri- and post-trigger deflections plotted against recording depth, where each cell pair is indicated by a specific color. Note that in each cell pair, two correlations could be measured (cell1 → cell2 and cell2 → cell1), hence two points of each color can be found at the same recording depth. Dashed lines are included in order to facilitate identification of the data that was derived from the same cell pairs in cases when the data values for the measured parameter differed substantially within the same cell pair. As the recording data for each cell pair was bootstrapped (see “Materials and Methods” section), each cell pair is represented by a range which corresponds to the 95% confidence interval of that parameter. In order to minimize overlap between neurons recorded at the same depths, we added a minor displacement in the depth axis for some of the recorded neuron pairs. **(B)** Similar plot for the corresponding peak amplitudes, expressed as net change in firing frequency divided by the baseline firing frequency.

For each pair of neurons recorded, peri-spike triggered time histograms (PSpTHs) were constructed based on the relative timings between each trigger spike (of the reference, or first, neuronal unit) and the response spikes (of the second neuronal unit) around that trigger point. PSpTHs provided a first-level overview of the correlation pattern between the two spikes, where the spike-firing correlation patterns were the focus of all analyses made. To further facilitate the analysis of such correlation patterns, we also made Kernel Density Estimations (KDEs) of the spike activity of the second spike in relation to the occurrences of the first spike (spike-triggered KDE, SpT-KDE; Figures 1C,D). For each cell pair, we calculated two SpT-KDE curves, one with Gaussian kernels with a standard deviation of 10 ms and one with a standard deviation of 1 ms. The coarser SpT-KDE was used for most of the analysis, but to better estimate the time and magnitude of triggered activity with sharp peaks, the SpT-KDE with a standard deviation of 1 ms was used. From these SpT-KDEs, we first defined a peri-trigger time window of activity deflection from the baseline (i.e., the deflection being closest to, and typically straddling, the trigger spike at time 0), a pre-trigger time window deflection (the deflection preceding the peri-trigger deflection and starting within 1 s or less before the time 0) and a post-trigger time window deflection (the deflection following the time 0 within 1 s or less). For each of these deflections, we defined an onset latency time, a time-to-peak, a duration and a peak amplitude of the frequency change from baseline activity, resulting in a total of nine measured parameters (Table 1, Figure 2).

**Table 1:** Summary of the quantified correlation parameters.

Parameter	Sample size	*P*-value	Mean value	Std dev	Coefficient of variation
Pre-spike deflection onset	42	*P* = 0.4	−220 ms	130 ms	59%
Pre-spike deflection duration	42	*P* = 0.5	140 ms	100 ms	71%
Pre-spike time of maximal deflection	42	*P* = 0.5	−140 ms	80 ms	59%
Peri-spike deflection onset	42	*P* = 0.3	−80 ms	50 ms	67%
Peri-spike deflection duration	42	*P* = 0.07	160 ms	90 ms	52%
Peri-spike normalized deflection height	42	*P* = 0.5	3.9	1.9	49%
Post-spike deflection duration	42	*P* = 0.3	160 ms	100 ms	61%
Post-spike time of maximal deflection	42	*P* = 0.5	140 ms	80 ms	59%
Post-spike normalized deflection height	42	*P* = 0.5	−0.37	0.22	59%

*Sample size indicates the number of unidirectional neuron pairs. The P-value given is the result of a Wilcoxon rank sum test for differences in median value between putative layer III and layer V neurons, based on subcortical recording depth. Note the high coefficient of variation (standard deviation divided by mean value), indicating that the parameter values were highly dispersed*.

All analysis of the PSpTHs was based on deflections from the baseline. Hence, to analyze deflections we first needed to define a baseline of activity. The baseline was defined as the average SpT-KDE for the time span from −1,800 ms to −500 ms and 500 ms to 1,800 ms, in relation to the trigger point. The onset and ending of the pre-trigger deflection was defined from the last (up to time zero) continuous depression (>40 ms) of the low-resolution SpT-KDE below the baseline (Figures 1C,D). The maximal absolute amplitude of the pre-trigger deflection was defined by detecting the minimum value of the depression. For the post-trigger deflection, the same procedure applied. For the peri-trigger spike activity, the onset was defined as the end-point of the pre-trigger deflection, and the end was defined as the onset of the post-trigger deflection. The peak amplitude, or deflection, for the peri-trigger spike activity was defined as the maximum value of the high-resolution SpT-KDE (1 ms Gaussian kernels) as these peaks could be too fast for the low-resolution SpT-KDE to capture.

**Figure 3:**
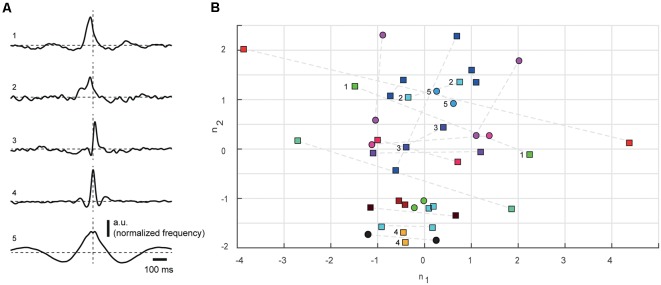
Multidimensional scaling (MDS) analysis of all nine parameters quantifying the correlation patterns for all cell pairs. **(A)** SpT-KDEs, normalized with respect to amplitude, for five different cell pairs (1–5), to illustrate the range of differences in the correlation patterns. Dashed lines indicate time 0 and baseline. Note that neuron pairs #3 and #5 were also shown in Figures 1C,D, respectively. **(B)** MDS of the parameters used for measuring the correlation patterns. As two correlation patterns were obtained for each cell pair, each pair is represented twice, where the same color indicates correlation patterns that were derived from the same pair. For triplet recordings, all pairs are indicated in the same color. Numerals refer to cell pairs shown in **(A)**. Cell pairs recorded below 0.9 mm, likely located in layer V, are indicated as circles. All cell pairs located above this level are indicated as squares. Dashed lines connect the two correlation patterns obtained from the same cell pairs. The stress value for the MDS plot was 0.1253.

In addition to plotting the data against depth in 2d-plots (Figure 2), we also displayed the data in the 9-dimensional space defined by the nine measured parameters of the correlation patterns using multidimensional scaling (MDS; Figure 3B). Since some parameters were measures of amplitude while others were measured in seconds, they were normalized to avoid specific parameters from having disproportional weight in the resulting MDS analysis. All values were normalized to the mean of the absolute value for each parameter across the neurons. For instance, the first amplitude of the pre-trigger deflection for each cell was divided by the mean of the absolute value of that parameter for all cells. The MDS plot thus represents the Euclidean distances between the normalized values of the nine parameters measured. The collapse of the 9D-space to the 2D-space was done using the Matlab function cmdscale (2016a, Mathworks), and had the stress value 0.1253.

Note that the ECoG pattern of all our preparations followed the classical pattern of ketamine-xylazine anesthesia, i.e., it was associated with episodes of synchronized ECoG intermixed with episodes of desynchronized ECoG (Chauvette et al., 2011). Each correlogram was an average across all of these conditions. Hence, the differences in brain states within the time period of a neuronal recording can be expected to have been larger than the average differences between preparations.

We also made a comparison between the correlation patterns during ongoing tactile afferent stimulation patterns vs. the spontaneous activity outside time periods of ongoing stimulation. To identify neurons that responded to the peripheral stimulation, we compared the activity in a time window before the onset of the tactile afferent stimulation pattern (1,000 ms of pre-stimulus activity) with the activity during the ongoing peripheral stimulation (350 ms of post-stimulus activity, as the stimulation patterns lasted up to 350 ms). The indicator of the presence of an evoked response was that the post-stimulus time window contained activity that for at least four consecutive time bins (at a bin width of 10 ms) exceeded two standard deviations of the activity in the pre-stimulus time window. To calculate the standard deviation of the pre-stimulus activity, we used the pre-stimulus part of the KDE of the entire peri-stimulus time window (the responses of all eight stimulation patterns were pooled), and the post-stimulus part of the same KDE was checked for responses exceeding two standard deviations. In 13 of the 21 cell pairs explored, the responses passed this threshold criterion and were hence considered in this part of the analysis.

### Statistics

We used the Wilcoxon rank sum test to evaluate the differences in median value between putative layer III and layer V neurons for all the quantified measures of the spike firing correlation patterns (Table 1).

The cross-correlograms represent an average of the correlation between the two spikes of the neuron pair recorded. In order to test whether the quantified deflection parameters of the cross-correlograms (see above) differed systematically between neurons, we used bootstrapping to resample the data 100 times for each cell/parameter. The resampled data were used to identify the 95% confidence intervals, which were displayed for the selected parameters (Figure 2).

**Figure 4:**
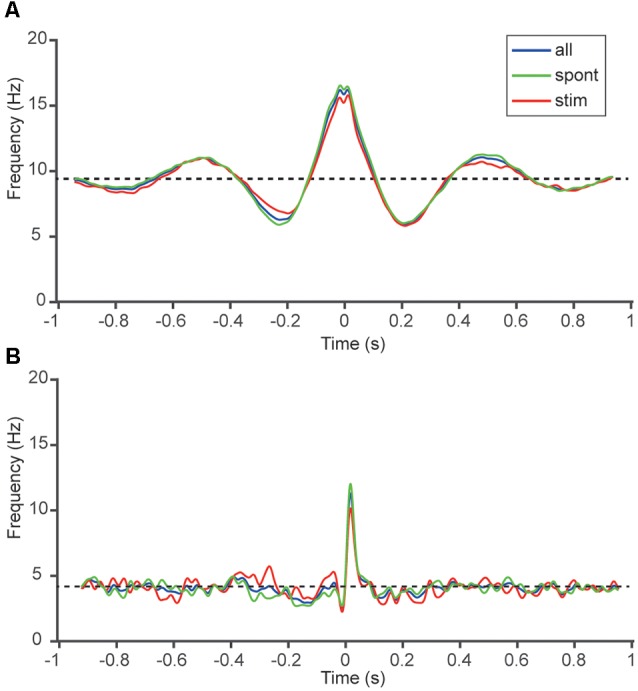
Correlation patterns altered only marginally with ongoing tactile afferent input. **(A)** SpT-KDEs for the same sample cell pair as in Figure 1C. The three traces represent the SpT-KDEs of the spiking activity without tactile afferent stimulation (green), during tactile afferent stimulation (red) and the two periods combined (blue). The Pearson correlation between spontaneous and stimulated spike activity was 95%. **(B)** Similar display for the sample cell pair in Figure 1D. The Pearson correlation between spontaneous and stimulated spike activity (green and red curves) was 44%.

Correlation coefficients between SpT-KDE-curves obtained during spontaneous activity and during stimulation patterns (Figure 4) were calculated using Pearson correlation coefficient.

## Results

Using a previously described technique for dual neuronal recordings from single patch pipettes in the extracellular recording mode (Bengtsson and Jörntell, 2009; Bengtsson et al., 2013), we recorded a total of 13 neuron pairs and three neuron triplets in the S1. Each triplet can be combined to yield three unique neuron pairs. However, in one of the triplets, all three neurons were not active simultaneously, so only two unique neuron pairs could be formed from that triplet. The other two triplets generated three unique neuron pairs each. Therefore, in total, we obtained 21 unique neuron pairs. In each such pair, the correlations between the two neurons could be quantified in either direction by switching the trigger spike. Hence a total of 42 cell pair correlations were studied. In each of these pairs, the recorded cells were classified as being pyramidal cells on the basis of an absence of spike bursts longer than three spikes, an overall low firing activity, and the rare presence of fast spike bursts (interspike intervals of less than 5 ms) typically of 2, more rarely of 3, spikes (Luczak et al., 2009).

Figure 1A illustrates an example of a triplet recording. As previously described (Bengtsson and Jörntell, 2009), even near-simultaneous occurrences of two separate spikes are possible to identify as the signal simply becomes the sum of the waveshapes of the coincident spikes (Figure 1A, bottom). Figure 1B illustrates the principles of the template-based method for spike identification, which were adapted to reliably capture all visually identifiable occurrences of each identified spike type of the recording. The main scope of this article was to identify the patterns of correlation between the spikes of each cell pair, or their cross-correlation. This was made during the full duration of the recordings, i.e., they include spontaneous activity and activity evoked by spatiotemporal patterns of skin stimulation to the tip of digit 2 where the condition with ongoing peripheral stimulation constituted less than 20% of the total recording time (we explore whether there were any differences between the correlation patterns during spontaneous and evoked activity towards the end of the “Results” section). The average firing frequencies of the individual cells ranged between 0.5 and 44 Hz. However, here we focused on the analysis of the temporal shape of the correlation patterns, which in principle is uncoupled to the firing frequency. Figures 1C,D illustrate how such cross-correlations could appear, in raw PSpTH of the second spike, and in SpT-KDEs. SpT-KDE resembles spike-triggered histograms, but instead of outputting the distributions across a set of time bins, it forms a continuous function. The continuous function consists of a convolution of Gaussian kernels centered over each spike time.

As shown in Figures 1C,D, the typical SpT-KDE profile contained a peak of activity surrounding the trigger spike and a decrease in activity before and after the trigger spike. Gaussian kernels with a standard deviation of 10 ms were used to estimate the baseline activity and the points where the spiking activity deflected from the baseline activity before and after the trigger spike, respectively. To estimate the time and magnitude of the peri-spike peak activity, where the peak from the PSpTH could be estimated to last for less than 20 ms, we instead used SpT-KDEs with a standard deviation of 1 ms for the analysis of the peak. A smaller standard deviation in the SpT-KDE brings a higher temporal resolution but an increased susceptibility to sampling errors, which is why this signal appears as a much noisier representation of the underlying PSpTH. But, as can be seen in Figure 1D, the SpT-KDE with the higher temporal resolution (standard deviation of 1 ms) is better at capturing fast activity changes in the PSpTH around the trigger spike. Therefore, we used the faster SpT-KDE to identify the sharper peaks of the correlation patterns that occurred around the trigger spike. Figure 1 also illustrates that the spike firing correlation patterns could be symmetric (Figure 1C) or asymmetric, with an excentric peak and/or pre-post-trigger deflections with different depths or latency times (Figure 1D). That the peaks in the correlation patterns between two nearby cortical neurons can be centric or excentric has previously been shown (Denman and Contreras, 2014; referred to as “zero-spanning” or “offset” correlation peaks in that article). However, here we analyzed a number of additional quantifiable parameters (Table 1) in the correlation patterns that could be used to compare the correlation patterns of different cell pairs.

Figure 2 illustrates the peak time points and the peak changes in frequency for the pre-, peri-, and post-trigger events for each neuron pair, plotted against recording depth. In each panel, each neuron is represented by its 95% confidence interval, as calculated by a bootstrapping procedure, for the illustrated parameter. Note that the values are dispersed widely along the x axes and essentially non-overlapping between neurons at the 95% CI. Also, in cases of overlap between two neuron pairs for one parameter, they rarely overlapped for any other parameter. This suggests that the correlation patterns were widely different between the different pairs. Note also that there was essentially no relationship between recording depth and the values of any of these parameters. Most of the neurons were recorded within a relatively narrow range of 0.55–0.75 mm, likely corresponding to layer III (de Kock and Sakmann, 2009). Another group of neurons was located around 1.0 mm, likely corresponding to layer V (de Kock and Sakmann, 2009). Table 1 summarizes the main properties of the correlation patterns across the population and quantifies the statistical differences between neurons of the two main groups of neurons based on depth. Notably, the high coefficient of variation for each parameter value indicates that there is a large variability in the correlation patterns within the population of neuron pairs. The absence of a statistical difference between putative layer III and layer V neurons moreover indicates that there was no particular feature in the correlation patterns that was dominant in either group of neuron pairs.

In agreement with the wide variation in the measured parameters, the correlation patterns of different cell pairs were non-homogeneous and appeared to be widely different between neuron pairs (Figure 3A). To better summarize this observation for the population of cell pairs, we used a MDS analysis of the distribution of all parameters measured (Table 1) for the correlation patterns (Figure 3B). Hypothetically—to give an example—in the population of neuron pairs recorded, it could be that if the pre-trigger onset latency is short, it is associated with a central peak of high amplitude and short post-trigger onset latency. If the correlations between a set of neuron pairs showed this type of recurring patterns, whereas the correlations between other neuron pairs did not, the MDS would be expected to display some degree of clustering for this set. However, if this set of neuron pairs differed substantially with respect to the other parameters, they would not cluster in the MDS, and consequently, this would be compatible with an absence of repeatable correlation patterns across the population. For the MDS, we calculated a vector from the correlation pattern of each neuron pair, based on the four parameters for the deflection from the baseline activity: onset of deflection, duration of deflection, time to peak and normalized spiking frequency at the point of maximal deflection. Concatenating the parameter values from the pre-, post- and peri-trigger deflections results in 9-parameter values, or a 9-dimensional space in which each neuron pair was located. Classical MDS was used to reduce the dimensionality so that this 9-dimensional space could be plotted on the 2D plane. As the dispersion of the parameter values for the cell pairs in both groups of neurons that is present in Figure 2, Table 1 is also present in Figure 3B, this suggests that few repeated patterns in the spike-time correlations existed and that each pair of neurons could be unique in terms of its correlation pattern.

As all of our recordings were long-term recordings of spontaneous activity mixed with intermittent tactile afferent inputs, a question that arose was if the correlation patterns observed were somehow influenced by the input to the cortex evoked by the stimulation. Because the tactile afferent inputs consisted of stimulation patterns lasting up to 350 ms (Oddo et al., 2017), separated by stimulation pauses of up to 1.5 s, we could analyze correlation patterns within and outside periods of active tactile afferent inputs. Only neurons classified as actually responding to this tactile afferent input was included, hence this analysis comprises 13 of our 21 unique cell pairs. Figure 4 shows the SpT-KDE for two different neuron pairs, where the SpT-KDEs of the time periods without active stimulation (spontaneous) and of the time periods with active stimulation are compared. Notably, there was little difference between the correlation patterns under these conditions, suggesting that the main features of the correlation patterns between a pair of neurons remained stable whether the spikes were recorded during spontaneous activity or during periods of active peripheral input. In Figure 4A, the Pearson correlation between the SpT-KDEs was 95% whereas in the cell pair in Figure 4B the Pearson correlation was 44%. In the population of pairs recorded (*N* = 13 unidirectional correlations), the average Pearson correlation was 56 ± 27%.

## Discussion

Using single electrode, dual neuronal spike recordings, and a set of quantifiable parameters, we found a wide range of correlation patterns between pairs of adjacent neurons in S1 neocortex. Most of the cell pairs were located at depths corresponding to layer III (de Kock et al., 2007; de Kock and Sakmann, 2009) and were putative pyramidal cells. This suggests that the spike activity correlations for adjacent neurons of the same layer identity do not follow a uniform pattern. Although direct synaptic communication between adjacent neocortical neurons is not uncommon (Petersen and Crochet, 2013; Jouhanneau et al., 2015) most of the correlation patterns analyzed here occurred outside the time range expected for monosynaptic communication between the two cells of each pair. This suggests that the correlation patterns were primarily shaped by the afferent input the two neurons in each pair received. A conclusion that can be drawn is therefore that between pairs of adjacent neocortical neurons of the same layer identity, there are wide variations in the pairwise activity differences of their physiologically effective afferent synaptic input.

### Methodological Issues

A relevant question is if the correlation patterns we observed under anesthesia would remain in the awake state. The question is related to the question if the pathways that information travels through the neocortical circuitry changes with anesthesia. The information that does exist on this issue is that ketamine anesthesia does not seem to alter the recruitment order of groups of local cortical neurons (Luczak and Barthó, 2012). This would suggest that the correlation patterns could look similar in the awake state, even though one may expect that anesthesia results in a generally lower level of activity in the neocortex (Constantinople and Bruno, 2011). This interpretation seems to gain some support from our observation that the correlation patterns remained largely unchanged under peripherally evoked activity compared to spontaneous activity (Figure 4; see further discussion below).

### Relationship to Previous Studies of Correlation Patterns Between Adjacent Neocortical Neurons

Previous studies of paired neocortical neuronal recordings of nearby neurons have been based on multi-electrodes with electrode separation of more than 200 μm (Ghoshal et al., 2009; Denman and Contreras, 2014) and are hence not directly comparable with the present study in terms of the distances between the two neurons of the pair. Furthermore, these previous studies primarily focused on the correlations in the trial-to-trial variation in overall spike counts and on the spike synchrony around time zero to quantify the differences between such pairs. In the present study, we instead identified a number of quantifiable parameters by which the correlation patterns could be compared statistically, and found that the individual cell pairs as a rule differed from each other across multiple such parameters (Figure 2).

### Factors Influencing Correlation Patterns in Spike Firing

The shape of the correlation pattern between two neurons will depend on a number of factors and it is possible that relatively small, but systematic, differences between two pairs can create large differences in the correlation patterns between different cell pairs. The factors that can be expected to influence the correlation patterns can roughly be divided into two categories, regional and local. A possible regional factor would be differences in the spatiotemporal activity of the afferents that drive for example the layer III neurons. Such differences in spatiotemporal afferent activity could conceivably also occur within subregions of the S1 cortex, which would explain our findings of different pairs of layer III neurons having different correlation patterns. If regional differences in the spatiotemporal patterns of spontaneous afferent activation occur, as our findings would imply, then such differences should be traceable for example to consistent dissimilarities in the shapes and/or frequencies of spontaneous up-states measured intracellularly (Poulet and Petersen, 2008). No evidence for such systematic differences exists, but appears not to have been actively sought for so far. Important examples of local factors are the overall spike firing activity or excitability, i.e., the difference between the membrane potential and the spike firing threshold of the neuron, and differences in weighting of the synaptic input between the two neurons of a pair. In either case, a possible interpretation of our findings is that the physiological neocortical network structure would not be canonical, as even differences in the shapes of spontaneous afferent activity would indicate differences in intracortical connectivity to neurons of the same type. There might also exist remnants of an underlying early developmental canonical microcircuitry that has gradually been modified by network shaping due to learning.

### Models of Synaptic Organization in Neocortex

Specific types of neurons, as defined by their layer location, have been shown to have common features in their intrinsic membrane dynamics that distinguish them from other neuron types (de Kock et al., 2007), and the intrinsic properties of the synaptic connections have been proposed to be layer dependent (Lefort and Petersen, 2017). Such observations are supplemented with observations that a particular type of neuron will primarily receive synaptic input from specific sets of other neuron types (Jiang et al., 2015; Reimann et al., 2015). From this line of reasoning, it would be expected that neurons of the same type would share similar afferent inputs. A possible consequence of this view, which can be regarded a bottom-up approach to understand the synaptic organization of the neocortex, is that the correlation patterns between pairs of neurons of the same type would be expected to be similar.

In what can be regarded as top-down approaches to the same question, previous studies have indicated that adjacent neurons provided by the same input (Oddo et al., 2017), or recorded in the same behavioral situation (Gawne and Richmond, 1993; Reich et al., 2001), tend to have differentiated responses. This is compatible with the view that the neocortex *in vivo* appears to strive to differentiate the firing of its constituent neurons (Renart et al., 2010) within the constraints of the statistically dominant patterns of local afferent connectivity.

An alternative interpretation is that neurons in each layer are further differentiable into additional subtypes. Indeed, based on transcriptomics, several recent studies suggest that layer V neurons can be subdivided into very large number of subgroups (Tasic et al., 2018). Such transcriptomic subgroups have been proposed to have different patterns of afferent and efferent connectivity (Economo et al., 2018), which would be compatible with our findings if one assumes that each recorded cell pair was a unique combination of neuron subtypes according to transcriptomics. Hence, one interpretation of our results could be that early genetic programs are responsible for shaping the local transcriptomics, which in turn could lead to the differences in connectivity our data suggests. However, although statistical differences in afferent connectivity between neurons of different transcriptomic subtypes are demonstrable, the differences are relatively small (Kim et al., 2015), such that the differences between individual neurons within a subtype might well be larger than those between the subtype populations. A question that arises is whether differences in transcriptomics are a cause or a consequence of individual neurons having different types of afferent inputs and/or efferent targets. Differences in learned synaptic weights amongst a common set of afferent inputs would make the neurons experience different temporal patterns of synaptic inputs, which could lead to an activity-dependent regulation of their transcriptomics (Hrvatin et al., 2018).

### More Detailed Considerations of Synaptological Organization

The dissimilar correlation patterns between different pairs of neocortical neurons are likely a consequence of the extent to which the local afferents providing effective synaptic inputs to the different pairs of neurons are concerted in their activation. In agreement with this line of reasoning, London et al. (2010) found that activation of single spikes into one neuron in a recorded population of local neocortical neurons could increase firing probability in other neocortical neurons up to more than 100 ms after the occurrence of the spike, although with the maximal probability increase occurring within the first few ms after the spike. The long latency effects can be interpreted as if the perturbation induced by the single spike will be transmitted through polysynaptic routes in the cortical neuronal network. To explain the dissimilar correlation patterns we observed here, a possible interpretation is that different layer III neurons, for example, tap off input from various positions in such chains of synaptically connected neurons. The correlation patterns between two neurons would hence depend on which specific parts of this chain they are tapping off their input from.

The synaptic input to neocortical neurons is expected to arise from other cortical neurons to 80%–85%, and only 15%–20% from thalamocortical afferents (Bruno and Sakmann, 2006). Previous studies of the neocortex indicate that spontaneous waves of excitation (up states) and excitation evoked by external input are associated with the same neuronal recruitment order (Luczak et al., 2009, 2013; Luczak and Barthó, 2012), which could explain the high degree of similarity in the correlation patterns, or the robustness of these correlation patterns, across conditions with solely intrinsically generated excitation vs. the situation with a presence of an externally driven excitation (Figure 4). These observations also suggest that the structure of the afferent cortical network has a larger influence on the synaptic input patterns to a neuron than external sources of input to the network. Hence, the correlation patterns in the spontaneous activity of two neurons are likely to reflect the position each neuron in the pair has in the cortical, rather than in the thalamo-cortical network.

Previous studies of the activity correlation between pairs of neocortical neurons have had more focus on the magnitude of the correlations (Lampl et al., 1999), and how it can change for example with brain state (Poulet and Petersen, 2008), but did not further analyze the temporal patterns of the correlations. Our data suggest that the correlation patterns are highly variable, even among neuron pairs of the same type, as defined by their layer location. Our findings hence argue for a largely heterogeneous connectivity within the neocortex, where the organization of synaptic inputs is more specific to the individual neuron than to the neuron type. A possible interpretation of our findings is that adjacent neurons of the same type, probably through their intrinsic plasticity mechanisms, instead learn to differentiate their synaptic inputs among the pool of afferent fibers that they both receive. This would lead to a differentiation of the response patterns of adjacent neurons, which we have previously also reported being the case for the processing of tactile inputs (Oddo et al., 2017) and which is hence supported by more general observations on the de-correlated firing relationships between neocortical neurons *in vivo* (Gawne and Richmond, 1993; Reich et al., 2001; Renart et al., 2010).

## Ethics Statement

All animal experiment procedures in the present study were in accordance with institutional guidelines and were approved in advance by the Local Animal Ethics Committee of Lund, Sweden (permit ID M118-13).

## Author Contributions

HM and HJ designed the study. JN, HM and HJ wrote the article. JN, JE and AW performed the experiments. HM, JN, JE, AW and HJ made the analysis.

## Conflict of Interest Statement

The authors declare that the research was conducted in the absence of any commercial or financial relationships that could be construed as a potential conflict of interest.

## Abbreviations

ECoG: electrocorticogram
KDE: kernel density estimation
MDS: multidimensional scaling
PSpTHs: peri-spike triggered time histograms
S1: primary somatosensory cortex
SpT-KDE: spike-triggered KDE.
